# Treadmill-based task for assessing spatial memory in head-fixed mice

**DOI:** 10.1016/j.xpro.2021.100770

**Published:** 2021-08-21

**Authors:** Jake T. Jordan, Kelsey D. McDermott, M. Agustina Frechou, Matthew Shtrahman, J. Tiago Gonçalves

**Affiliations:** 1 Dominick P. Purpura Department of Neuroscience and Gottesman Institute for Stem Cell Biology and Regenerative Medicine, Albert Einstein College of Medicine, Bronx, NY 10461, USA; 2 Department of Neurosciences, University of California San Diego, La Jolla, CA 92093, USA

**Keywords:** Microscopy, Model Organisms, Neuroscience, Behavior

## Abstract

Several mouse *in vivo* neuronal recording techniques require head fixation. Head-fixed treadmill walking can be used to design tasks that enable the study of neural activity in the context of behavior. Here, we provide a detailed protocol for constructing a treadmill with tactile spatial cues, training mice on a rewarded behavioral task, and analyzing behavioral data. We discuss common problems and solutions we have developed to optimize training. Finally, we demonstrate how to test spatial memory performance using this task.

## Highlights

• Protocol for a rewarded head-fixed treadmill task for mice
• Assembly of treadmill and overview of a surgical procedure
• Training of mice on head-fixed, rewarded spatial memory tasks
• Analysis of reward search by spatial analysis of lick behavior along a treadmill

## Graphical abstract

**Figure:**
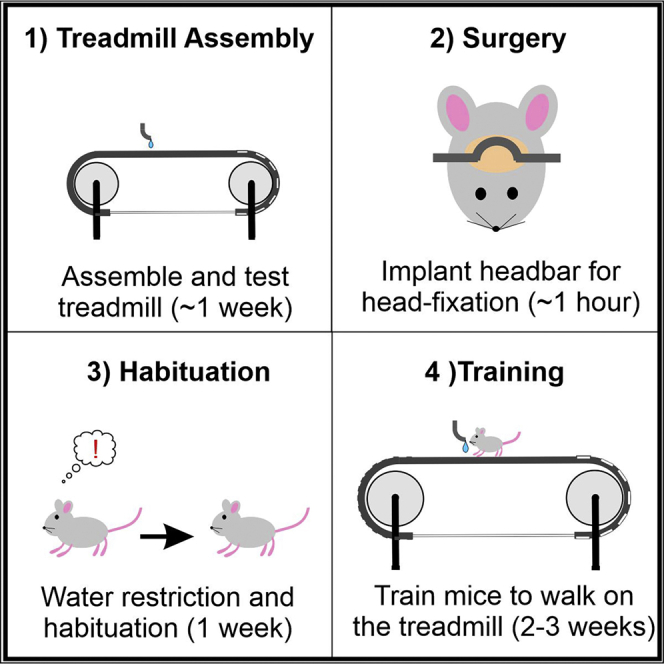

## Before you begin

Prior to beginning, obtain institutional animal use approval and follow legal and ethical guidelines for animal care and use throughout.

The protocol was developed using male and female C57Bl6J mice bred in house and aged 12–13 weeks at the time of surgery were used for these experiments. It can also be used with other strains of mice and has been successfully tested using 18-month-old mice. Animals were housed on a 14/10 h light-dark cycle with experiments performed during the light phase. Laboratory chow was available *ad libitum*. Except during citric acid water treatment, tap water was available *ad libitum*. All procedures were approved by the Institutional Animal Care and Use Committee (protocol #: 00001197).

This protocol describes steps for assembling a head-fixed, animal-driven treadmill for mouse behavior (Royer et al., 2012; Danielson et al., 2016) as well as instructions for training the mice and analyzing their behavior. Although similar set-ups that use virtual reality (VR) environments exist, this task in particular is non-visual, relatively easy to learn and can thus be used to train mice with visual or mild cognitive impairments, such as aged or certain transgenic mice. The treadmill consists of a fabric belt wrapped around two foam wheels on ball bearings. Four radio frequency identification (RFID) tags are attached at regularly spaced intervals along the belt and an optical rotary encoder measures the spin of one of the treadmill wheels. The position of the mouse is determined by converting the output voltage of the rotary encoder to the mouse’s velocity between RFID reads. The treadmill belt can occasionally slip on the wheels, introducing an error in the estimation of the position but using several RFID tags at different points along the belt will ensure that this error does not accumulate. Placing RFID tags at transition points between tactile zones also allows for comparisons of behavior and/or neural activity across zones as well as at transition points. An additional RFID tag is placed at a location along the belt where the mouse will receive a reward of sweetened water. These water rewards are dispensed from a lick port equipped with a sensor to measure licking behavior. Spatial learning and memory can be assessed by analyzing the spatial distribution of licking behavior.

### 3D-printing and machining of custom parts

**Timing: 1–2 days**

Detailed information on the design and assembly of each component is available on the lab GitHub Page: https://github.com/GoncalvesLab/HeadFixedTreadmill.

1. 3D print the platform used to hold the mouse up during treadmill walking.
2. Custom-made titanium headbars are implanted on the mouse’s skull to hold it in place during recordings. They can be laser-cut from ∼1–1.5 mm thickness titanium sheet stock and re-used indefinitely.
3. The head-restraint assembly is made from metal components for maximum stability and durability. Custom metal components (head plate, brackets, etc.) should be cut by an experienced machinist.
4. The treadmill wheels are made from an exercise roller cut into 9 cm-wide cylinders.
  a. As precisely as possible, drill a hole through the center (∼4.5 cm from the edge) and insert one of the axle rods through the center. The wheel (foam roller segment) should be in the middle of the axle rod.
  b. To prevent slippage of the foam wheel against the axle, apply cyanoacrylate glue to the sides of the wheel where the rod is protruding out.

### Treadmill assembly

**Timing: 1 week**

**Figure 1:**
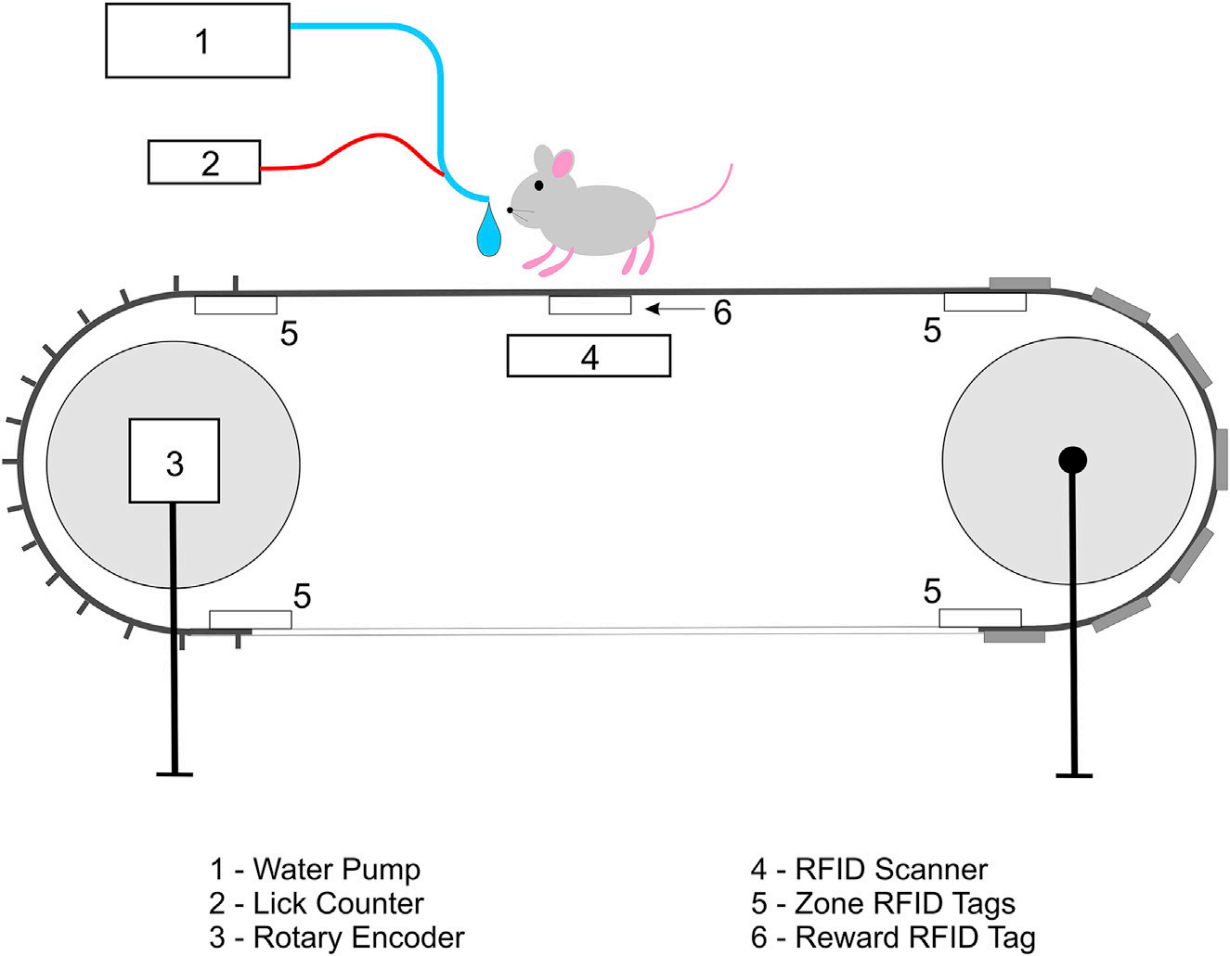
Electronic components of head-fixed treadmill apparatus

**Figure 2:**
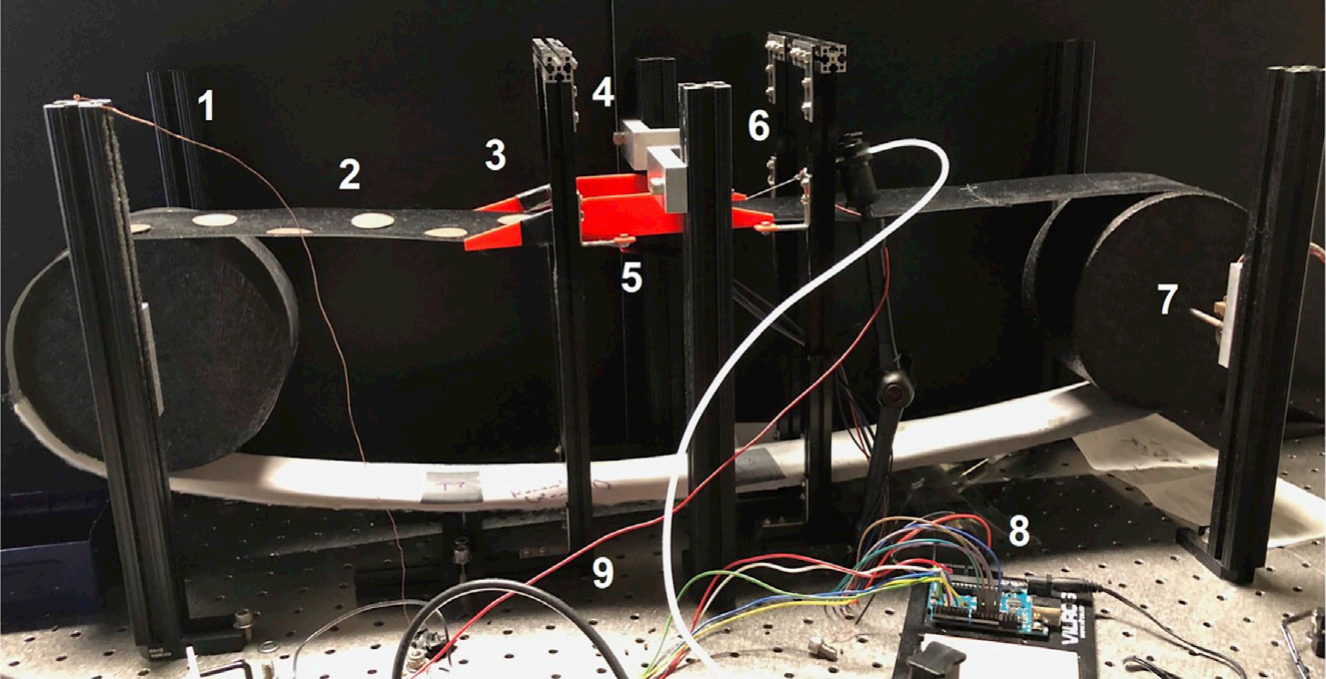
Assembled treadmill with a 180-cm-long belt 1 – Grounding copper wire; 2 – treadmill belt; 3 – mouse platform; 4 – head plate; 5 – RFID scanner affixed to the bottom of the platform; 6 – reward port; 7 – optical rotary encoder; 8 – RFID Arduino; 9 – red cable is input from reward port to the lick counter (not pictured). Positioning of all Arduinos, lick counter, pump and NI box is not critical and can be determined by the experimenter’s preference.

The treadmill apparatus consists of several electrical components used to collect data about mouse movement on the treadmill and licking behavior as well as to trigger and administer water rewards (Figures 1 and 2).

5. Setup the data acquisition computer. Experiments are controlled by a PC equipped with a data acquisition card (National Instruments PCIe-6323).
  a. Be sure that the computer used has MATLAB (version 2020a or newer) installed with the scripts RunTreadmill.m and OpenTreadmillFile.m stored in a directory listed in the path variable.

***Note:*** We are acquiring treadmill data and controlling reward administration using NI hardware and MATLAB software, however other data acquisition systems can also be used, including open-source alternatives based on eg. Raspberry Pi.

6. Insert axle rods into the ball bearings (Figure 3).
  a. One axle will require the wheel of the optical rotary encoder to be attached before it is inserted into the ball bearings.
    i. To do this, gently remove the wheel and encoder from the plastic casing so as not to damage the wheel or encoder (the plastic casing can be damaged and discarded).
    ii. The encoder will then be attached to custom ball bearings plate with matching screw holes.

**Figure 3:**
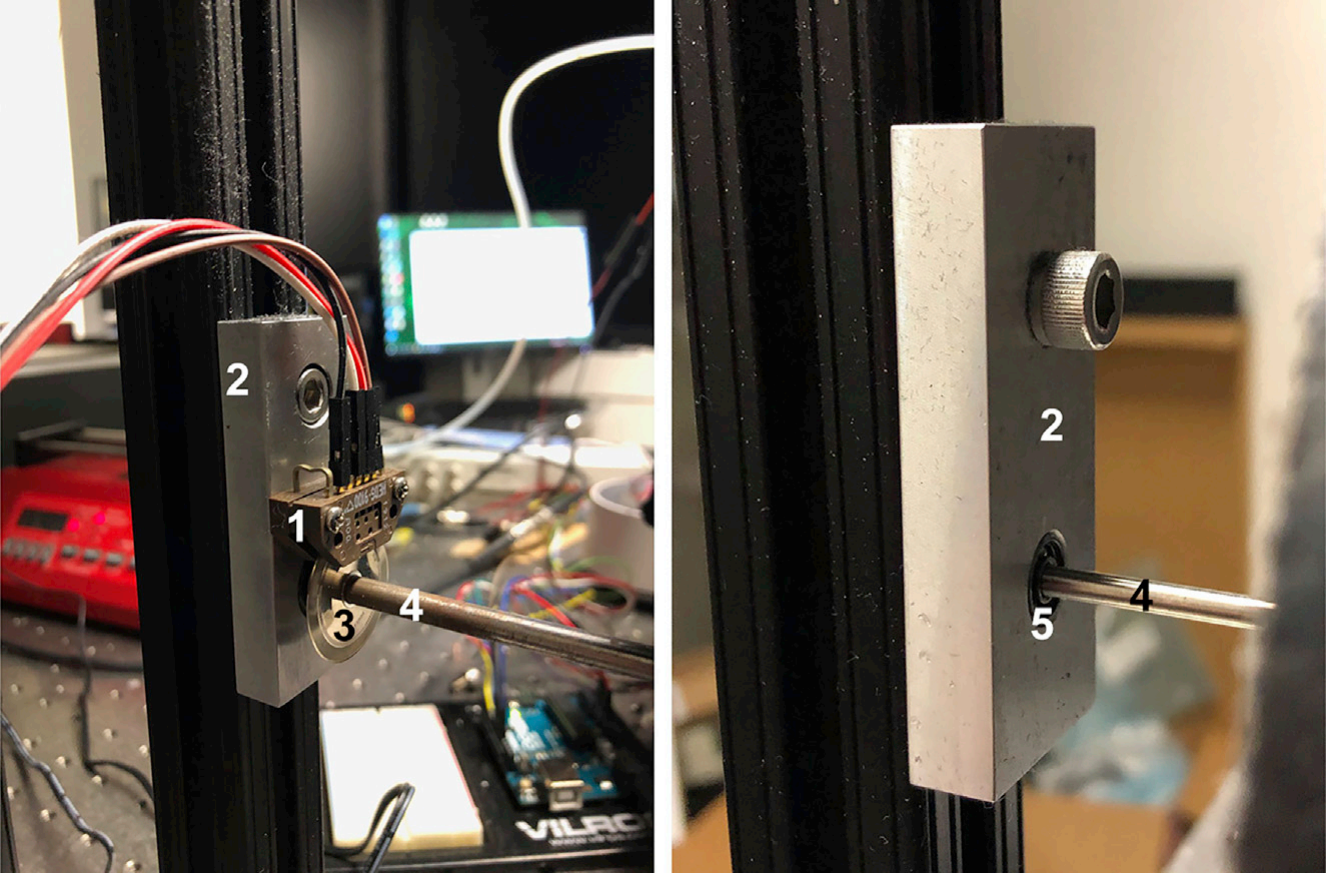
Assembly of wheel axle with ball bearings plates Left: The optical rotary encoder (1) is attached to one of the custom plates (2). The encoder disk (3) is attached to an axle (4) which is inserted into the ball bearings. Right: An axle inserted into the ball bearings (5) on the three other custom plates.
7. Assemble the treadmill wheels.
  a. For our purposes, we have extruded 1-inch aluminum rails attached vertically to an air table (Figure 2). These rails form the frame that is used to suspend the wheels, axles and ball bearing plates as well as the platform and RFID reader.
  b. The two rails used to suspend each wheel should be spaced approximately as far apart as the two ball bearings plates on either side of the axles assembled in step 6.
  c. The two suspended wheels should be spaced approximately 50 cm apart from each other with the platform in the middle.
8. Assemble the platform with RFID scanner attached.
  a. We affix the RFID scanner to the bottom of the platform using heavy duty electrical tape directly under where the mouse will be walking.
    i. The wires to the RFID scanner should be soldered and individually isolated with heat-shrink tubing, as the vibrations caused by mouse walking can lead to short-circuiting and malfunction.
  b. The height of the platform should be slightly (∼0.5 cm) lower than the top of the two wheels.
9. Attach the head plate to the head plate brackets and attach the brackets to a pair of rails so that the head plate is approximately 2.5 cm above the platform.

**CRITICAL:** (Step 9): Mice may have difficulty running on a surface that is too smooth or be distracted by a surface that is very soft. Pay attention to how mice react when you begin their training, and reassess the quality of the belt and its texture if they have trouble pulling the belt or tend to remain in one area instead of walking (after some training).

10. For the treadmill belt, cut the velvet fabric into 7.5 cm-wide strips. The total length of our belt is 180 cm which can be made from four 45 cm-long strips of velvet appended together. This can be done quickly by stapling the strips together, but they may also be sewn together. Do not close the belt into a ring until it wrapped around both wheels (see step c below).
  a. The mouse needs to be able to grip the belt early in training to begin walking. This can be done by using variable tactile cues (e.g., for a spatial memory task) or uniform tactile cues (e.g., if studying non-spatial memory). Sandpaper circles of different grit or stripes made with a hot glue gun can serve this function.
  b. Attach the RFID tags to the underside of the belt with a piece of duct tape.
    i. We use 5 tags: 4 for tactile zone transitions and 1 for a reward site.
  c. Wrap the belt around the treadmill wheels and platform and append the ends of the treadmill belt together (stapled or sewn).
11. Solder a wire to a metal gavage needle. This wire will be plugged into the lick counter. Add the reward port by assembling the gavage needle, tubing and 10 mL syringe. Place the syringe in the clamp on the microinfusion pump and follow manufacturer instructions to program 10 μL pulses. Use a flexible arm clamp to place the reward port near where the mouse will be walking such that it can be near the mouse’s snout during trial but moved away for ease of access when attaching or removing mice.
12. The rotary encoder and RFID reader are operated with Arduino microcontrollers. Plug wires and BNC cables into the appropriate components following the instructions on our Github (https://github.com/GoncalvesLab/HeadFixedTreadmill).
13. Test the treadmill by running a short (∼1 min) test session using the RunTreadmill.m program in MATLAB. Confirm that water rewards are being administered and run the OpenTreadmillFile.m script and follow the instructions at the top of that code to confirm that data from all treadmill components are being correctly acquired.
  a. Do this at the beginning of every training day or immediately after changing treadmill belts or reward locations.
14. For synchronizing the movement, lick and reward data with neuronal recordings (imaging or electrophysiology), generate a transistor-transistor logic (TTL) signal from the recording apparatus and record it by running the wire to an input of the National Instruments data acquisition hardware at the beginning and end of each recording session, or for each frame. For example, many microscopes can output a “FrameOut” signal for each recorded video frame. This signal can be used to synchronize the behavioral data with neural recordings.

***Note:*** The treadmill belt does not necessarily need to be made of the same material listed in the key resources table. Whichever material is used will need to be light enough to be easily pulled by the mouse, but must also not slip on the foam wheels. Belt slippage (i.e. when the belt and wheel are not turning at the same rate) will cause an error in estimating the position of the mouse using the output of the optical rotary encoder.

### Preparation for surgery

**Timing: <1 hour**

15. If using the Janelia lick counter, mice will need to be implanted with a grounding pin soldered to a small tungsten wire.
  a. To assemble, clamp the gold pin in a helping hand alligator clamp. Insert a tungsten wire into the shallow hole of the flat end of the pin. Solder and then snip the wire approximately 1 mm from the pin. Likewise, solder a female connector to a long piece of wire (long enough to connect the mouse’s implanted pin to the lick counter) to ground the mouse. Licks will be sensed when a grounded mouse contacts the lick port, closing the circuit.
16. Prepare the surgery area using sterile techniques. For this surgery, in addition to the headbar and grounding pin, you will need:
  a. Scalpel
  b. Forceps
  c. Dental curing light for Optibond and Flow-It Accelerated Light Cure (ALC) composite adhesives.

***Note:*** Although the Comparator Dual Port Lick Detector requires a grounding pin, not all lick counting systems require this, a possible alternative is to use an optical lick counter. We chose to use the Comparator Dual Port Lick detector because we had previous experience with this system.

## Key resources table

**Table:** 

REAGENT or RESOURCE	SOURCE	IDENTIFIER
**Chemicals, peptides, and recombinant proteins**		

Artificial sweetener	Splenda	n/a
Citric acid	Milliard	MIL-CTRCACD-5-A
Isoflurane, USP	Covetrus	11695-6777-2
Carpofen	Zoetis	1041283
Optibond	Kerr	33381
Flow-It ALC Flowable Dental Composite	Pentron	N11H
Dental cement powder	Lang Dental	1330
Dental cement liquid	Lang Dental	1306

**Deposited data**		

All code and sample data are available on our GitHub page	Gonçalves Lab GitHub	n/a

**Experimental models: Organisms/strains**		

C57BL6J mice	Jackson Laboratory	000664

**Software and algorithms**		

MATLAB	MathWorks	2020a
Arduino IDE	Arduino	1.8.12
RFID library for Arduino	Arduino	MRFC-522 Library

**Other**		

Computer	Dell	Optiplex 7080
Arduino Uno	Arduino	A000066
Arduino Due	Arduino	A000062
Data acquisition card	National Instruments	PCIe-6323
Breakout box	National Instruments	BNC 2090A
Data cable	National Instruments	SHC68-68-EPM
Microinfusion pump	Braintree Scientific	Braintree Scientific BS-8000
Lick port	Janelia Research Campus	Comparator Dual Port Lick Detector
Optical rotary encoder	Digi-Key	HEDS-5500#A05
RFID scanner	SunFounder	RC522
RFID tags (5)	Amazon	ISO14443A
Wires	Amazon	120 piece kit
BNC cables (3)	Amazon	MC2791
Heat-shrink tubing	Amazon	560 piece set
Flexible arm (to hold and move lick port): Articulating Magic Arm (11 inch long)	Amazon	Mmo-PN-329618834
Gold pin (1 per mouse)	a-msystems.com	520200
Gold pin connector	a-msystems.com	520100
Copper wire		
Velvet textured treadmill material	McMaster-Carr	88015K1
Custom mouse platform	See GitHub	n/a
Custom headbar (1 per mouse; reusable)	See GitHub(laser-cut by IMH products IMH.com)	n/a
Custom head plate	See GitHub	n/a
Custom head plate brackets (2; 1 left, 1 right)	See GitHub	n/a
Custom ball bearing plates (3)	See GitHub	n/a
Custom ball bearing plates with encoder screw holes (1)	See GitHub	n/a
Exercise roller (cut into 3.5 inch cylinders)	ProsourceFit	High density cylindrical roller 18” (L) x6” (diam.)
Axles (2)	McMaster-Carr	60355K501
Ball bearings (4)	McMaster-Carr	1257K86
10 mL Syringe (20G x 1.5”) (at least 3)	Becton Dickinson	309604
Gavage needle (for lick port)	Pet Surgical	2018-08
Tubing	CM Scientific	R-3603
T-slotted aluminum rails (6; 30.5 cm)	McMaster-Carr	47065T503
T-slotted framing fasteners (6)	McMaster-Carr	47065T142
Headbar screws (2)	McMaster-Carr	92196A052
Forceps	Fisher Scientific	08-953C
Scalpel (No. 3 handle)	Fisher Scientific	13-812-234
Sterile scalpel blades	Fisher Scientific	22-079-684

## Step-by-step method details

### Surgery

**Timing: <1 h for headbar implant only, 1–2 days if including a viral injection and cranial window implant**

Here, we describe how to attach the headbar to the mouse skull as well as implant a grounding pin for lick counting. For details on intracranial viral injections and/or cranial window implants, which should be done prior or simultaneously to headbar implantation, see Mizrahi et al. (2004) and Dombeck et al. (2007).

**Figure 4:**
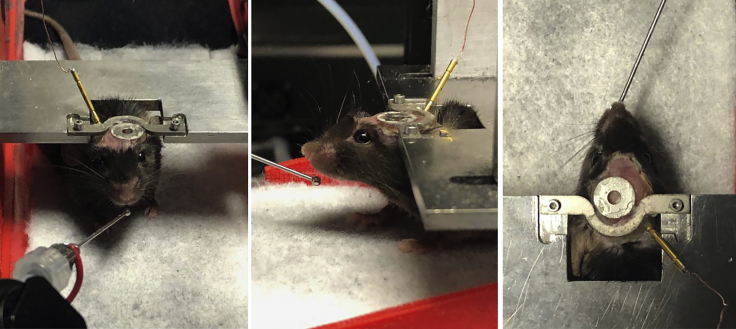
Three views of a mouse with a surgically implanted cranial window and headbar, head-fixed to a custom head plate Note the lick port positioned by the spout and a grounding wire connected to an implanted pin.

1. Anesthetize the mouse in accordance with IACUC-approved protocols.
2. Once anesthetized and unresponsive to touch, maintain anesthesia with 1%–2% isoflurane and 0.5 L/min O_2_ with the mouse on a heating pad.
3. Clean scalp with betadine and 70% ethanol. Use a scalpel to make a longitudinal incision about 5–6 mm long, exposing the skull.
4. Using scalpel, thoroughly etch the surface of the skull to enhance the bonding of the Optibond and dental cement to the surface of the skull. Then, apply a layer of Optibond dental adhesive over the exposed surface of the skull. If necessary, pull the skin back with forceps to avoid getting Optibond on the skin. Cure the Optibond using blue light.
  a. If injecting virus and/or implanting electrodes or a cranial window, follow that procedure now and then return to this protocol.

5. If necessary, sterilize grounding pin and implant on the back of the skull, over the cerebellum.
  a. Prepare a smooth portion of the back of the skull by trimming away neck muscle and then drill a small (∼1 mm) craniotomy.
    **CRITICAL:** The muscle has a tendency to bleed, so carefully scrape away the connective tissue where the muscle connects to the skull with a scalpel to minimize bleeding.
  b. Using forceps with a steady hand, insert the tungsten wire end of the gold pin into the craniotomy and hold as still as possible. With the other hand, apply Flow-It ALC around the base of the pin and then cure with blue light.
    **CRITICAL:** Make sure to implant the pin at an angle (Figure 4) that will not interfere with the treadmill setup when you attach it to the mouse or with the objective if you are imaging.

The blue light used to cure the composite dental adhesives may irritate the mouse’s skin (e.g., ears) when exposed. Aim the light away from it, or cover up any exposed skin when curing.

6. Implant the headbar using dental cement.
  a. Apply a thin layer of dental cement over the entire surface of the skull. This layer should have a low viscosity (determined by relative amounts of dental cement powder and dental cement liquid mixed together) to allow it to spread easily.
  b. After 2–3 min, apply a small amount of thick dental cement to the back of the skull. Immediately place the headbar in the middle of this thick spot of cement so that the wings of the headbar are perpendicular to the long axis of the mouse’s body. Be sure to balance the headbar before the cement dries fully so that the mouse will not be at an angle while head-fixed.
  c. Apply one last coat of dental cement over the back of the headbar and spread around to the rest of the skull with care not to cover the wings of the headbar.
7. Allow the dental cement 5–10 min to dry.
8. Inject the mouse subcutaneously with 5 mg/kg of carpofen dissolved in saline (at a concentration of 1 mg/mL)and remove from anesthesia.
9. Mice should begin to recover from anesthesia within minutes and will be fully responsive in approximately one hour.

### Water restriction and habituation

**Timing: 1 week**

In order to ensure mice are sufficiently motivated to walk on the treadmill for water rewards, water consumption must be restricted in some way that follows IACUC-approved water deprivation protocols. We replace homecage water with 2% citric acid water, which reduces water intake and is a safer alternative to removing homecage access to water completely (Reinagel, 2018; Urai et al., 2021).

10. Replace homecage water with 2% citric acid water.
  a. Weigh mice prior to citric acid water treatment (baseline) and then every day for 5 days and then once per week after treatment has begun.

**CRITICAL:** Ensure that animal weight does not drop below 85% of the baseline weight. If this does happen, immediately allow access to 1 mL of clean water. If a mouse continues to be below this threshold, remove from experiments.

11. During the first week of citric acid water treatment, habituate mice to handling by the experimenter in the room in which experiments will be conducted. Do this for at least 3 min per day for 3 days (does not need to be consecutive).
12. Prior to starting any behavior session, ensure that there is sufficient water in the syringe and that all treadmill data is being acquired.
  a. To test whether all data are being acquired, run a test session
13. One week after the beginning of citric acid water administration, begin training.

### Treadmill training

**Timing: ∼2–3 weeks**

Mice will vary in the amount of training sessions needed to walk well on the treadmill. Begin with 20-min training sessions, and reduce the training time as mice improve walking by 5 min per session until training session are 10 min long. Define the number of laps you will need for your experimental purposes, and train mice until the entire cohort has reached that level or has been excluded.

14. Run the program RunTreadmill.m and enter all the relevant information in the GUI.
  a. Do not start the data acquisition until the animal is head-fixed.
15. Affix the headbar to the head plate (Figure 4).
  a. If the mouse turns so that its rear legs are to the side or nearly in front of the head, gently tug the treadmill belt until the mouse is in a walking position. Some mice may continue to do this during the first couple of days of training, but it will diminish over time.

**CRITICAL:** When placing mice on and off the treadmill, take care not to stress them as this will impair performance. Quickly transfer mice from the cage to the treadmill platform and avoid dangling them by the tail. Do not overly force the mouse into head-fixation. Place the mouse behind the head-fixation stage. Many mice naturally duck their heads under the head-fixation stage to investigate. When they do this, simply pull the belt forward lightly so that the headbar is now near the fixation point and hold the headbar in place while screws are inserted. This minimizes the degree to which you have to force the mouse in place. Over training they will become more used to this process and tend to voluntarily walk to the head-fixation position.

16. On day 1, train the animal to lick the reward spout by administering a free 10 μL reward (10% Splenda in water) and touching it to their snout.
  a. Only do this once per day at the beginning of the training session for the first 2–3 days.
17. Run the session from the MATLAB command line.
18. Start a timer for the duration of the session. Turn off the room lights and come back when the timer has gone off.
19. Unscrew the headbar and gently place the mouse back in the homecage.
20. Check the performance on the task (e.g., rewards earned, licking behavior, etc.) by running the OpenTreadmillFile.m program.

***Note:*** Reward volumes vary in the literature (4–20 μL). Although we use a 10 μL reward, others may decide to vary this amount based on their specific experimental needs.

***Note:*** For sanitary purposes, clean the syringe containing sweetened water regularly (once per week) by flushing with 70% ethanol and tap water to avoid mold growth. Regularly clean the area of mouse feces and replace treadmill belts when they become soiled. This will vary based on frequency and volume of use, but is usually around once every 1–2 months.

## Expected outcomes

**Figure 5:**
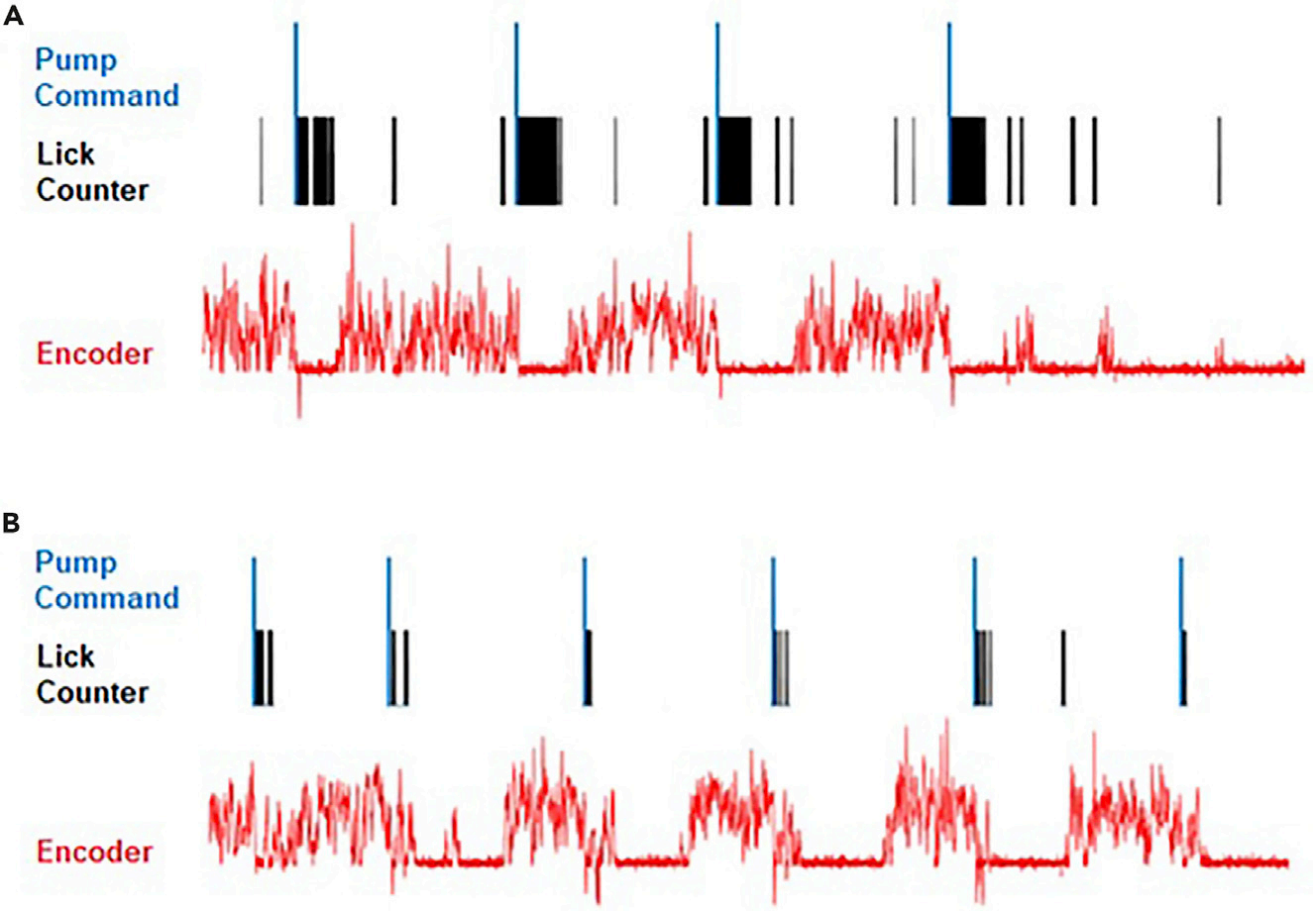
Example treadmill output data Data from example sessions of a well-trained mouse receiving sucrose water at the reward site (A) and the same mouse during a probe trial in which reward was omitted (B). Blue traces represent the voltage input commands that would normally trigger reward (pump is disconnected during probe trials, black ticks represent licks, and red traces represent the output of the rotary encoder.

The recorded data structure contains the output of the rotary encoder (Treadmill belt movement), licking behavior and reward triggers (Figure 5) as well as scans of each RFID tag.

**Figure 6:**
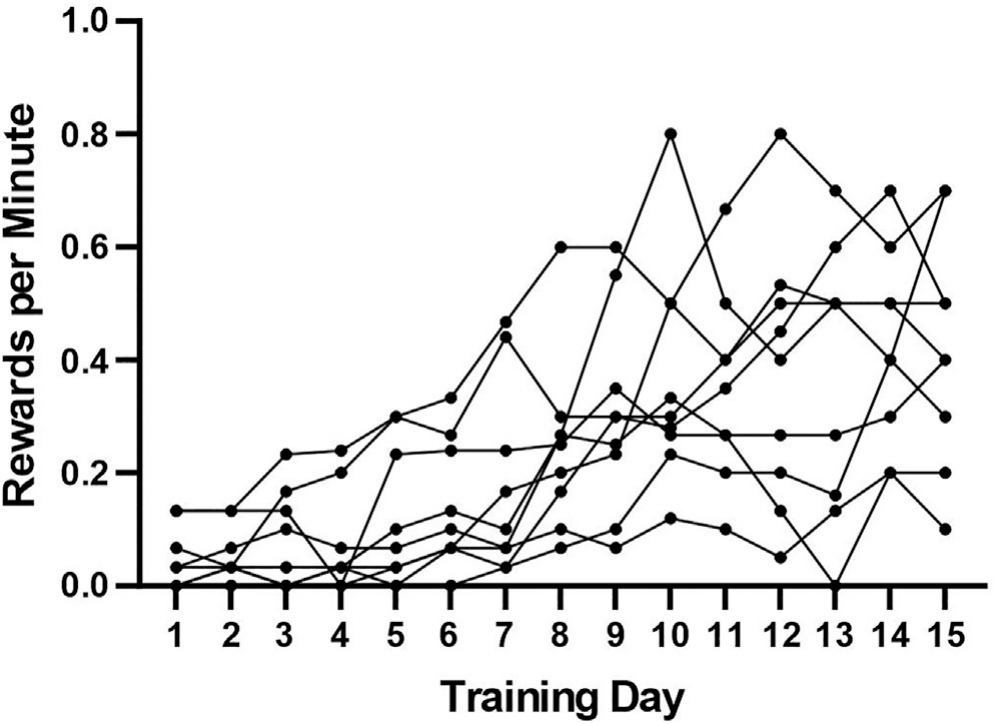
Example of 9 mice trained on the treadmill task for 5 days per week for 3 weeks

Mice should learn the task in around 2–3 weeks, with the majority of licking occurring at or near the reward zone. Completed laps per session and overall lick rate will generally be low in the beginning of training and will gradually increase. Trained mice have ranged from 2–26 laps per 10-min session. Some experiments may require mice to run a certain number of laps within a certain amount of time and therefore some sessions may fail to reach this threshold, however, this number is likely to vary across experiments and, if applicable, neural recording techniques (see Figure 6 for performance of an example cohort from the beginning of training).

## Quantification and statistical analysis

**Figure 7:**
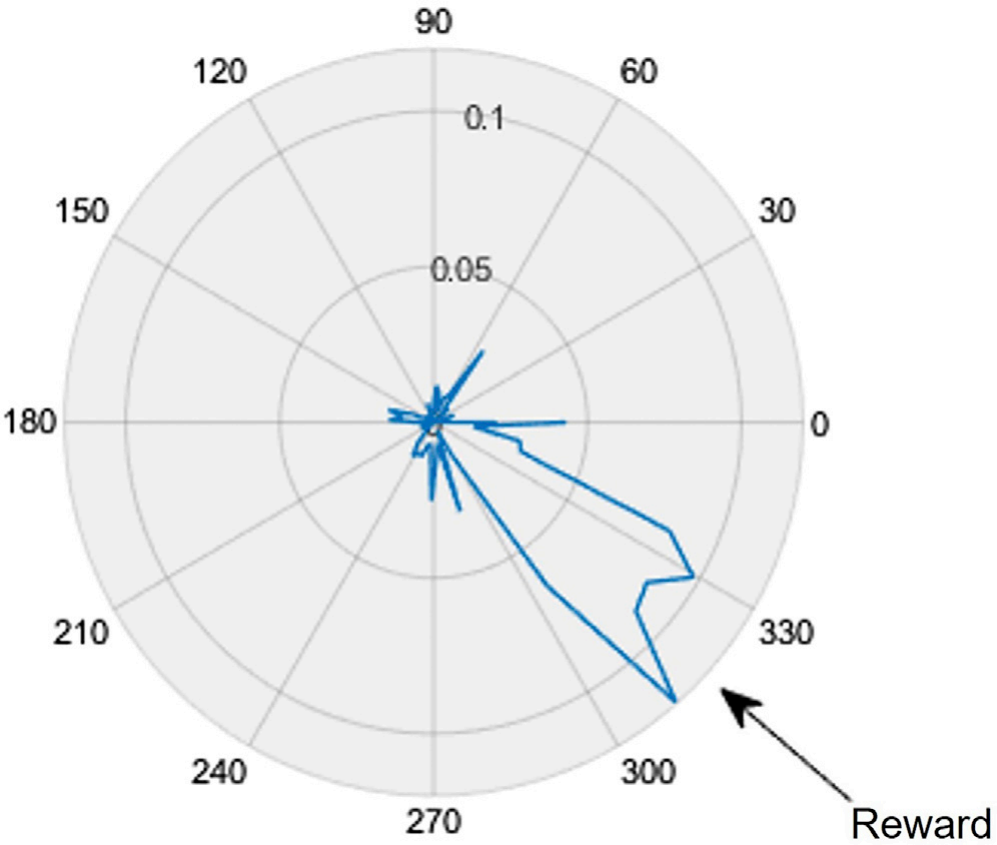
A normalized lick rate map of a session run on a 180 cm treadmill The blue trace indicates the fraction of licks occurring in 3 cm wide bins. A clear preference is shown to lick in vicinity of the reward location.

Analysis of spatial licking behavior can be done using our custom software. Typically, learning can be demonstrated by a gradual increase in the fraction of the total licks occurring in the rewarded quadrant of the belt. “Lick maps” can also be computed to help visualize the spatial specificity of lick behavior by dividing the belt into bins of a certain length (e.g., 3 cm) and computing the fraction of all licks occurring in each bin (Figure 7).

Spatial memory can be tested by conducting a probe session in which reward is omitted. This removes the possibility that the presence of water in the lick port or any sounds from the reward pump is indicating to the mouse where a reward may be administered. Thus, mouse must rely on memory of the rewarded location to direct licking behavior. To conduct a probe trial, switch off the reward pump and then run a session as you normally would. Overall lick rates will decrease sharply during these sessions as the mice do not get rewards. However, licking behavior will still be concentrated near the reward zone. An alternative to probe trials is to make the reward administration contingent on the detection of licking within the reward location.

## Limitations

Our head-fixed system prohibits studying the contribution of head movement to neuronal activity, although a new device has recently been developed to overcome this limitation (Bakhurin et al., 2020). This is an important limitation to consider for hippocampal studies that may want to analyze head-direction cells and other similar cell types.

Probe sessions, in which the pump is off and no reward is given (Figure 7), can be used to measure lick preference for reward locations. However, these sessions can only be performed 1–2 times per cohort before animals give up on the task entirely.

## Troubleshooting

### Problem 1

Mouse movement can be enough to cause issues such as false lick detection from the lick counter or disconnection of the RFID scanner. If the scanner is temporarily disconnected, it will malfunction and shut off mid-session (steps 18 and 10).

### Potential solution

To prevent false lick detection, we reduced the sensitivity of the lick counter by soldering a 47 nF capacitor at pins IN1 or IN2 and GND (on the bottom of the board). To prevent scanner disconnection, we soldered all wires to the scanner and then carefully insulated each wire with heat shrink tubing at the soldering site.

### Problem 2

Early on in training, some mice will grab the lick port with their front paws as they lick. This will yield a very long pulse that (using our analysis software) will be counted as a single lick, despite many licks occurring (steps 18 and 20).

### Potential solution

This behavior usually goes away with training. If it becomes a recurrent issue, the experimenter may want to put a barrier around the lick port to prevent grabbing. It is always good practice to check the lick data after each session to screen for these types of issues and potentially exclude animals.

### Problem 3

Mice that are small in size due to age or genetic line may have more difficulty pulling the treadmill belt. This could lead to them learning the task more slowly, or result in fewer laps (step 18).

### Potential solution

If possible, use adult mice that are >20 grams so that they can pull the belt easily. If smaller mice are necessary, we recommend trying out different textures to find ones that are easiest for the mice to grip (sandpaper and felt have worked well for us). Additionally, the level between head fixation and belt can be adjusted so that smaller mice have better posture and do not have to stretch.

### Problem 4

Mice do not perform many laps, even with weeks of training (steps 16–20).

### Potential solution

In addition to habituation by the experimenter prior to training, it is recommended that the experimenters avoid stressing mice as much as possible. For instance, chronic drug administration with repeated restraint and injections should be avoided and alternative strategies for drug administration should be followed. Mice should be handled as gently as possible to avoid stress.

If headbars are implanted at an angle so that the mouse’s head is fixed at an unnatural angle during walking, it will likely not ever perform the task. During surgery, ensure that the headbar is attached at an angle consistent with the mouse’s natural head angle during walking.

### Problem 5

Lap sizes computed by the custom analysis software are inconsistent (step 20).

### Potential solution

This is usually caused by the mechanics of the treadmill, e.g., slippage of the belt on the wheel with the optical decoder. If the belt and decoder are not rotating together, then distance computed for each lap (demarked by RFID scans) will be inconsistent and can affect spatial analysis of behavior or neural activity. Make sure that the belt is not wrapped too loose around both wheels. There should be some slack of the belt on the underside of the treadmill, but it should not be dragging on the table. Another possible source is resistance in wheel axles. Check the axles by spinning the wheels and ensure that the rotate smoothly. If not, realign the axle and ball bearings until there is no resistance.

### Problem 6

Mice walk backwards or move the belt back and forth rapidly to trigger water rewards (step 18).

### Potential solution

Older systems have used single reward tags which when scanned, trigger a reward. Mice can “cheat” this system by moving the single tag over the scanner rapidly, triggering many rewards without having to complete laps. Our system is designed to prevent this from occurring by requiring a preceding RFID tag to be scanned prior to the reward RFID tag being scanned to trigger reward. I.e., the mouse has to run forward through the reward zone in order to trigger the reward.

### Problem 7

RunTreadmill.m is freezing and/or rewards are being delivered many seconds after the reward RFID tag is scanned (step 17).

### Potential solution

This has occurred when using version 2019b of MATLAB. Switching to version 2020a resolved this issue.

## Resource availability

### Lead contact

Further information and requests for resources and reagents should be directed to and will be fulfilled by the lead contact, Tiago Gonçalves (tiago.goncalves@einsteinmed.org).

### Materials availability

No new reagents were used for this study.

## Data Availability

Custom code is available on the Gonçalves lab GitHub Page: https://github.com/GoncalvesLab/HeadFixedTreadmill.
